# Amelioration of right spatial neglect after visuo-motor adaptation to leftward-shifting prisms^☆^

**DOI:** 10.1016/j.cortex.2009.06.002

**Published:** 2010-03

**Authors:** Janet H. Bultitude, Robert D. Rafal

**Affiliations:** Wolfson Centre for Clinical and Cognitive Neuroscience, School of Psychology, Bangor University, UK

## 1 Introduction

Visuo-motor adaptation to rightward prismatic shifts reduces signs of left spatial neglect on a wide range of measures (Rossetti et al., 1998; Tilikete et al., 2001; McIntosh et al., 2002; Pisella et al., 2002; Berberovic et al., 2004). As there are hemispheric asymmetries in spatial attention mechanisms, it may be useful to examine whether prism adaptation can produce similar improvements in neglect of the *right* hemispace following left hemisphere damage. We report improvement in a patient with mild right spatial neglect following adaptation to leftward-shifting prisms.

## 2 Participants and methods

### 2.1 Participants

Participants were one patient with right spatial neglect (female, age = 75 years, left-handed) and eight right-handed, neurologically healthy age- and gender-matched control participants (Mean age = 73.4 years, Standard Error of the Mean – SEM = .82).

Patient DS was hospitalised with unintelligible speech, left gaze deviation, right neglect, right facial weakness and hemiplegia with brisk reflexes on the right and bilateral Babinski signs. A Computerised Tomography (CT) scan revealed a large left fronto-parietal haematoma due to haemorrhagic infarction.

Three months later, at the time of the current investigation, DS was referred to us after her speech therapist noticed a tendency to leave the rightmost part of her workbooks uncompleted. At this time she had anomic aphasia with impaired repetition; however, her comprehension was relatively preserved and judged sufficient to enable informed consent. She had dense right hemiplegia and completed pen and paper tests for neglect with her left hand. She failed to copy the rightmost detail of a simple scene (Ogden, 1985); showed a mean 8.9% leftward line bisection bias on three lines (Wilson et al., 1987); and failed to cancel 2–3 rightmost targets on three cancellation tests (Gauthier et al., 1989; Edgeworth et al., 1998). A Magnetic Resonance Imaging (MRI) scan revealed a large left hemisphere lesion involving the frontal eye field, motor and premotor cortices, cingulate gyrus, posterior superior temporal gyrus and the parietal lobe (including the superior parietal lobule, precuneus, angular and supramarginal gyri).

### 2.2 Design and procedure

The effects of adaptation to leftward-shifting prisms on the neglect symptoms of DS were examined using a multiple baselines design, with eight testing sessions spanning eighteen days. Effects of both sham treatment (day 2) and prism treatment (day 7) were examined. In these sessions neglect was assessed using the Ogden copying task (Ogden, 1985), the Bells cancellation task (Gauthier et al., 1989), and a modified version of the line bisection subtest of the Behavioral Inattention Test (Wilson et al., 1987). Performance on the copying and cancellation tasks were at ceiling, therefore analyses focused on the results of the line bisection test. In each session DS bisected twelve 203 mm horizontal lines positioned to the left, middle or right of 4 sheets of A4. Bisection deviations from veridical were expressed as a percentage of line length, with negative values indicating a leftward bisection bias (right-sided neglect).

Comparison bisection data were collected from the healthy participants in three sessions completed on one day: at baseline, after sham adaptation, and after prism adaptation. Like DS, control participants used their left hands for all tasks.

During prism adaptation participants wore goggles containing adjustable wedge prism lenses that were set to induce no shift (‘sham’ treatment) or a 15° leftward visual shift (‘prism’ treatment). They made fifty pointing movements with their left hand, alternating between two targets positioned at eye level and arm's length 10° to the left and right of straight ahead. The goggles restricted the visual field such that participants received visual feedback of the second half of their pointing movement only. After touching each target, they returned their hand to the surface of the table in front of them.

Adaptation was confirmed by measuring an after-effect: Participants pointed to three targets located straight ahead and 10° to the left and right of body midline in a pre-determined pseudorandom order. A panel positioned under the chin occluded vision of the pointing arm. Pointing error was measured in degrees with the aid of markings on the underside of the panel, with negative numbers indicating leftward deviation. Twelve pointing measurements were taken in four sessions for DS (pre-sham, post-sham, pre-prism and post-prism), and in three sessions for the control participants (at baseline, post-sham and post-prisms).

## 3 Results

### 3.1 Prism adaptation

#### 3.1.1 Healthy controls

A repeated-measures Analysis of Variance (ANOVA) of pointing error with one factor, session (baseline, post-sham, and post-prism), revealed a significant main effect [*F*(2,14) = 14.45, *p* < .001] where mean pointing error was unchanged between the baseline (*M* = .9°, SEM = .67) and post-sham session [*M* = 1.9°, SEM = .72; *t*(7) = 1.12, *p* = .26] but shifted significantly rightward after prism adaptation (*M* = 4.9°, SEM = .55) compared to both baseline [*t*(7) = 5.37, *p* < .005] and post-sham [*t*(7) = 3.90, *p* < .01] pointing.

#### 3.1.2 DS

A repeated-measures ANOVA of pointing error with two factors, treatment (sham and prism) × session (pre and post), revealed a significant two-way interaction [*F*(1,11) = 21.9, *p* < .001] where pointing error was unchanged following sham treatment [Pre: *M* = 3.3°, SEM = .28; Post: *M* = 4.8°, SEM = .70; *t*(11) = 2.02, *p* = .07] but shifted 4.9° rightward following prism treatment [Pre: *M* = 5.6°, SEM = .55; Post: *M* = 10.5°, SEM = .35; *t*(11) = 8.48, *p* < .001].

### 3.2 Line bisection

#### 3.2.1 Healthy controls

There was a decrease in bisection errors across the baseline (*M* = −4.68, SEM = 1.53), post-sham (*M* = −3.66, SEM = 1.49) and post-prism sessions (*M* = −3.11, SEM = 1.44), although a one-way ANOVA revealed no main effect [*F*(2,14) = 1.63, *p* = .23]. Bisection errors were pooled across sessions and a 95% confidence interval around the mean confirmed a significant leftward bias [‘pseudoneglect’; CI_.95_ = (−5.54, −2.10)].

#### 3.2.2 DS

**Fig. 1:**
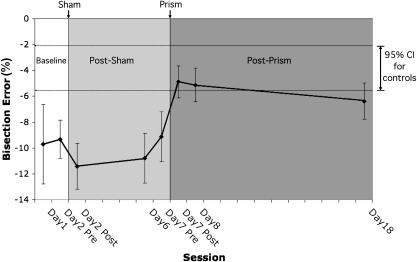
Average line bisection errors (±1 SEM) of patient DS for each session compared to the 95% confidence interval around the mean for healthy control participants. Sessions were grouped into three stages: baseline, post-sham, and post-prism as indicated by the shaded areas. Negative numbers indicate leftward bisection errors (right-sided neglect).

Fig. 1 shows DS's bisection performance across sessions compared to controls’. The sessions were grouped into three stages: baseline (day 1 and day 2 pre), post-sham (day 2 post, day 6 and day 7 pre), and post-prism (day 7 post, day 8, day 18). Bisection errors in all baseline and post-sham sessions were outside the 95% confidence interval for controls, but were within normal bounds in the first two post-prism sessions. A one-way ANOVA revealed a main effect of stage [*F*(2,93) = 7.49, *p* < .005]. *T*-tests revealed no difference between baseline (*M* = −9.52, SEM = 1.52) and post-sham performance [*M* = −10.44, SEM = .97; *t*(58) = .537, *p* = .593]. Bisection deviation in the post-prism stage [*M* = −5.46, SEM = .68] was smaller than both baseline [*t*(58) = 2.72, *p* < .01] and post-sham [*t*(70) = 4.20, *p* < .001].

Stability of improvement was evaluated with a one-way ANOVA, which showed no difference between bisection errors in the three post-prism sessions [*F*(2,33) = .45, *p* = .641], although there appeared to be a trend for a return to baseline. Mean error on day 18 was within one SEM of the 95% confidence interval around the mean for the control group, but was also not different from baseline [*t*(34) = 1.34, *p* = .19]. Five additional daily adaptation sessions administered after day 18 resulted in no further reduction in bisection error (*M* = −5.58, SEM = .80; not shown in Figure).

## 4 Discussion

To our knowledge this is the first report of a patient with right spatial neglect to be treated with prism adaptation. In previous work patients with left spatial neglect adapted to rightward- but not leftward-shifting prisms (Rossetti et al., 1998). We therefore used leftward-shifting prisms to treat this patient with right spatial neglect to induce a rightward orienting after-effect. Prism adaptation was effective both in inducing a rightward after-effect and in improving neglect as measured on a bisection task.

Healthy participants make small but systematic leftward errors in line midpoint estimations (‘pseudoneglect’; Bowers and Heilman, 1980). These shift rightward by 1–2% following adaptation to leftward-shifting prisms (Berberovic and Mattingley, 2003; Michel et al., 2003) but not always significantly so for manual bisection (Colent et al., 2000). The baseline bisection errors of DS were larger than the controls', and her post-prism error reduction of approximately 5% was greater than the shifts previously reported for healthy participants. These results indicate both the presence of mild neglect, and a reduction of bisection bias after prism adaptation beyond that which would be expected for controls.

Although further studies with greater numbers of patients are needed, our results suggest that the neurological process by which adaptation to rightward-shifting prisms ameliorates left neglect can occur in a similar fashion with leftward-shifting prisms for patients with right neglect. A proposed mechanism for the prism-induced improvements in left spatial neglect involves signals from right cerebellum that influence activity in the left parietal lobe via a network of left and right hemisphere areas (Pisella et al., 2006). These left parietal areas could be recruited for previously right parietal functions (Pisella et al., 2006), or may further influence the right superior parietal lobe via colossal communication (Striemer et al., 2008). Adaptation to leftward-shifting prisms using the left hand may result in a symmetrical process in patients with right spatial neglect, recruiting right parietal areas or influencing spared left hemisphere areas to restore rightward attention.

## References

[bib1] Berberovic N., Mattingley J.B. (2003). Effects of prismatic adaptation on judgements of spatial extent in peripersonal and extrapersonal space. Neuropsychologia.

[bib2] Berberovic N., Pisella L., Morris A.P., Mattingley J.B. (2004). Prismatic adaptation reduces biased temporal order judgements in spatial neglect. NeuroReport.

[bib3] Bowers D., Heilman K.M. (1980). Pseudoneglect: Effects of hemispace on a tactile line bisection task. Neuropsychologia.

[bib15] Colent C., Pisella L., Bernieri C., Rode G., Rossetti Y. (2000). Cognitive bias induced by visuo-motor adaptation to prisms: A simulation of unilateral neglect in normal individuals?. Cognitive Neuroscience.

[bib4] Edgeworth J.A., Robertson I.H., McMillan T.M. (1998). The Balloons Test.

[bib5] Gauthier L., Dehaut F., Joanette Y. (1989). The bells test: A quantitative and qualitative test for visual neglect. International Journal of Clinical Neuropsychology.

[bib6] McIntosh R.D., Rossetti Y., Milner A.D. (2002). Prism adaptation improves chronic visual and haptic neglect: A single case study. Cortex.

[bib7] Michel C., Pisella L., Halligan P.W., Luauté J., Rode G., Boisson D. (2003). Simulating unilateral neglect in normals using prism adaptation: Implications for theory. Neuropsychologia.

[bib8] Ogden J.A. (1985). Anterior-posterior interhemispheric differences in the loci of lesions producing visual hemineglect. Brain and Cognition.

[bib9] Pisella L., Rode G., Farnè A., Boisson D., Rossetti Y. (2002). Dissociated long lasting improvements of straight-ahead pointing and line bisection tasks in two hemineglect patients. Neuropsychologia.

[bib10] Pisella L., Rode G., Farne A., Tilikete C., Rossetti Y. (2006). Prism adaptation in the rehabilitation of patients with visuo-spatial cognitive disorders. Current Opinion in Neurology.

[bib11] Rossetti Y., Rode G., Pisella L., Farnè A., Li L., Boisson D. (1998). Prism adaptation to a rightward optical deviation rehabilitates left hemispatial neglect. Nature.

[bib12] Striemer C., Blangero A., Rossetti Y., Boisson D., Rode G., Salemme R. (2008). Bilateral parietal lesions disrupt the beneficial effects of prism adaptation: Evidence from a patient with optic ataxia. Experimental Brain Research.

[bib13] Tilikete C., Rode G., Rossetti Y., Pichon J., Li L., Boisson D. (2001). Prism adaptation to rightward optical deviation improves postural imbalance in left-hemiparetic patients. Current Biology.

[bib14] Wilson B., Cockburn J., Halligan P.W. (1987). Behavioural Inattention Test. Secondary Title.

